# Tumor Location Correlates with Clinicopathological Features and Prognosis of the Solid Pseudopapillary Neoplasm

**DOI:** 10.1155/2018/9023947

**Published:** 2018-07-02

**Authors:** Zibo Meng, Mingsi Cao, Feiyang Wang, Yushun Zhang, Heshui Wu

**Affiliations:** ^1^Department of Pancreatic Surgery, Union Hospital, Tongji Medical College, Huazhong University of Science and Technology, Wuhan 430022, China; ^2^Institute of Cardiology, Union Hospital, Tongji Medical College, Huazhong University of Science and Technology, Wuhan 430022, China

## Abstract

**Background:**

Solid pseudopapillary neoplasms (SPNs) of the pancreas are rare neoplasms with low malignant behavior. These neoplasms can be aggressive and cause bad ending of SPN patients. The purpose of this article is to identify certain prognostic factors.

**Method:**

We retrospectively evaluated 196 patients from our hospital and SEER database. We identified that tumor location was an independent prognostic indicator of SPN patients.

**Results:**

DSS and OS of pancreatic head SPNs (HOP) were significantly shorter than those of other locations (OOP). Operation methods and age were different between HOP and OOP groups. Compared to OOP group, patients in HOP group were younger. Operation time was longer, and hospital stays were longer.

**Conclusion:**

This work suggests that pancreatic head SPNs have distinct clinicopathological features and clinical outcome. It is urgent to optimize the treatment of SPN patients and identify effective prognostic indicators of SPN.

## 1. Introduction

Solid pseudopapillary neoplasms (SPNs) of the pancreas are uncommon, constituting 1%-2% of all pancreatic tumors [1]. It was first reported by Frantz in 1959 and classified as solid pseudopapillary tumors by the World Health Organization (WHO) in 1996 and redefined in 2010 [2]. SPNs are low, yet unpredictable, malignant potential tumors which always occur in young female [3]. The disease among most of the patients is localized, only 9–15% occurring with metastasis or local invasion [4]. At present, surgical treatment is the primary treatment for SPN. After complete margin-negative resection of the SPN, most of the patients are cured, with a low recurrence rate ranging from 2% to 9.4% according to several published SPN case series [5–10]. And the 5-year survival is favorable about 94%–97% [3, 11].

Although most SPN patients have excellent prognosis, some SPNs still exhibit malignant features, leading to recurrence and death of SPN patients. Articles reported that age, gender, tumor diameter, Ki-67 index, extrahepatic metastasis, complete resection of metastases, and recurrence might relate to the aggressive behaviors of the SPNs [12, 13]. However, the cases of SPN are relatively rare and above conclusions are based on small-sample case series and case reports. Reliable prognostic indicators need to be further identified and verified through large sample data analysis.

We know that different tumor sites of the same kind of tumor lead to distinct tumor invasion, relapse, and metastasis states. Khashab et al. report that the head location of pancreatic serous cystic neoplasms (SCNs), which are considered premalignant neoplasms, predicts the tumor aggressive behavior [14]. The degree of malignancy and slow progression between SPN and SCN are similar. Compared with other locations (OOP), the pancreatic head SPNs (HOP) have different clinicopathological features and clinical outcomes. Therefore, we collected 196 SPN cases to explore these clinical features of pancreatic head SPNs.

## 2. Materials and Methods

### 2.1. Patients and Data Collection

SPN cases were from our hospital and SEER database. From April 2012 to September 2016, 55 cases of SPN were diagnosed and received surgical treatment in our hospital. We retrospectively evaluated these patients and included all the eligible cases. Data including age, gender, race, symptoms, location, tumor size, surgical options, operation time, complication, number of lymph nodes removed, postoperative diet recovery time, postoperative hospital stay, total hospital stay, and survival information were extracted from medical records in our hospital. Survival period was measured from the time of diagnosis to the time of death or until January 15, 2018. This study was approved by the ethics committee of Union Hospital, Tongji Medical College, Huazhong University of Science and Technology. The written informed consent was obtained from all 55 participants in our institution.

For the cases from SEER database, we used SEER^∗^Stat software (version 8.3.4) to obtain the SPN cohort. We identified SPNs according to ICD-O-3 histology code (code: 8452). Patients were included if (1) diagnostic confirmation was positive histology or/and cytology; (2) SPN was the first tumor; (3) surgical procedure was applied; and (4) prognostic information was available. We extracted the available clinicopathological data which was mentioned above (including age, gender, race, location, tumor size, surgical options, number of lymph nodes removed, and outcome) from the SPN data list. Patients were followed up until December 2014. Since SEER data were from the public database, no ethical consent was needed for this part.

We divided the SPNs into the pancreas head originated group (HOP) and other parts originated group (OOP) in order to further analyze the clinicopathological features and prognostic character of the pancreatic head SPNs.

### 2.2. Statistical Analysis

We used chi-square test or Fisher's exact test to process qualitative variables. Two-sided Student's *t*-test or Wilcoxon test was applied to calculate the quantitative parameters. We applied Kaplan-Meier analyses with the log-rank test to examine the statistical differences in survival and portray the patient survival curves (disease-specific survival (DSS) and overall survival (OS)). We also used Cox proportional hazards regression analyses to calculate the hazard ratios with 95% CIs and to exhibit the effects on DSS and OS of the location of the SPNs. All *p* values were 2-sided, and *p* values < 0.05 were considered statistically significant. All the analyses were conducted using SPSS software version 20.0 (SPSS Inc., USA).

## 3. Results

### 3.1. Clinicopathological Features of Extracted SPN Cases

In total, 196 cases including 55 cases from our institution and 141 SEER cases were selected into our study. Most of the patients were female. The age of the patients ranged from 8 to 90 years (mean, 34.5 years) for all patients in two cohorts. In SEER group, 101 (71.6%), 16 (11.3%), 20 (14.2%), and 4 (2.8%) patients were white, yellow, black, and other races, respectively. All patients in our department were yellow. In our cases, 24 SPNs (42.9%) occurred in pancreatic head, 9 (16.1%) SPNs in pancreatic body, 11 (19.6%) SPNs in the pancreatic tail, and 11 (19.6%) SPNs were in other locations. Similar proportion was also shown in SEER cohort. The median tumor size was 5.0 cm and 5.4 cm, respectively, in our cases and SEER cases. We defined operation methods into four categories: OP1: local excision of tumor; OP2: partial pancreatectomy and partial or local pancreatectomy and duodenectomy without distal/partial gastrectomy; OP3: Whipple's procedure and extended pancreatoduodenectomy; and OP4: other operation methods. In total, 8 patients underwent OP1 procedure; 114 patients underwent OP2 procedure; and 40 and 30 patients, respectively, performed OP3 and OP4 surgeries. Mean number of resected lymph nodes was 8 (ranged 0 to 52) during the surgery. Clinical data of SPN patients in our hospital was more detailed. The most common initial presentation was found by accident (26/55, 47.3%), followed by abdominal pain (22/55, 40.0%), abdominal mass (5/55, 9.1%), and other symptoms (2/55, 3.6%). 31 patients (31/54, 57.4%) experienced postoperative complications including biliary fistula, infection, hemorrhage, intestinal fistula, and pancreatic fistula. Median operation time took was about 330 minutes, and restoration of oral feeding time was about 6 days. Median postoperative discharged time was about 12 days, and the total hospital stay was about 17 days. All the clinicopathological details were summarized in Table 1.

### 3.2. Disease-Specific Survival and Overall Survival for Different SPN Locations

**Figure 1:**
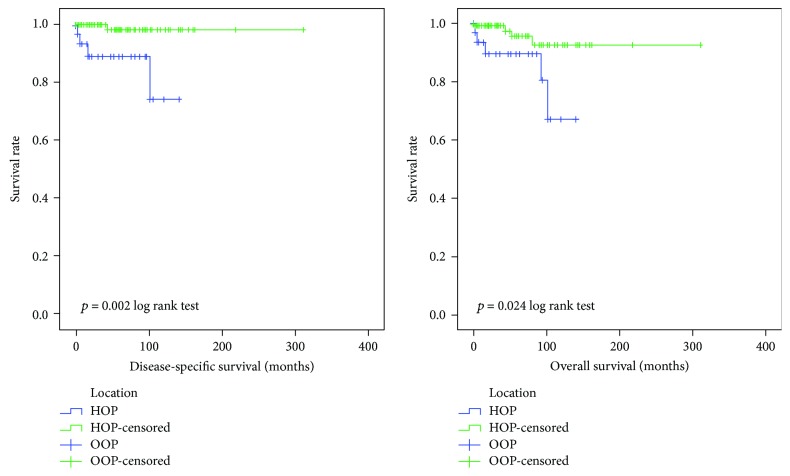
The DSS and OS K-M curves of SEER cohort according to the classification of location.

Survival information of pancreas head-originated SPNs were listed in Table 2. We analyzed survival data of 188 patients including 47 cases from our hospital and 141 SEER cases. The average follow-up time was 33.7 months in our department and 58.3 months in SEER database. In all patients, 5 cases suffered from SPN-related death and 14 patients died from all causes. In SEER cohort, 1-, 3-, and 5-year DSS rates were 98.5%, 97.8%, and 97.1%, respectively, and the 1-, 3-, and 5-year OS rates were 97.8%, 97.2%, and 95.7%, respectively. In our cohort, the HOP group had lower 1- and 3-year overall survival rates compared to the OOP group. For the small number of SPN patients in our hospital, we only used SEER cohort to analyze the prognostic factor.  Table 3 shows the prognostic factors of the DSS and OS. Multivariate analysis showed that only tumor location (*p* = 0.028) was statistically related to DSS. Tumor location (*p* = 0.022) influenced the OS of SPN patients. The DSS and OS K-M curves according to the classification of location are shown in Figure 1.

### 3.3. Comparison of Clinicopathological Parameters between HOP and OOP

Analyzed results are given in Table 4. Chi-square test or Fisher's exact test results showed that operation methods (*p* < 0.001) were statistically distinct between HOP and OOP in both cohorts. However, there were no differences between HOP and OOP in gender, symptom (*p* = 0.979), and complication (*p* = 0.667). Student's *t*-test or Wilcoxon test exhibited that age, operation time (*p* < 0.001), and hospital stay (*p* = 0.05) were different between HOP and OOP, while size, restoration of oral feeding time, and postoperative discharged time were not different between the two groups. We further investigated the data of 5 patients who died in our hospital; tumors of these patients had bigger sizes than the average level (mean size, 8.6 cm versus 6.1 cm). There was no significant difference in other data including ki67, *β*-catenin, vimentin, CD56, and CD10.

## 4. Discussion

SPNs are rare pancreatic cystic neoplasms which account for 1% of all pancreatic tumors and 3% of all cystic pancreas neoplasms [15]. With the continuous improvement of multidetector computed tomography (CT) technology, there has been a remarkable increase in the number of incidentally detected pancreatic cysts over the last decade [16]. It is not difficult to understand why the number of SPN cases reported throughout the literature was also increasing. Therefore, identifying SPNs that have the potential for malignancy seems particularly important. Considering the rarity of SPN, we collected 55 SPN cases surgically treated at our hospital and 141 SPN cases from SEER database to identify the potential factors which need to be considered when making treatment plan.

In our analysis, the majority of patients were young women, and SPNs often showed nonspecific symptoms, such as pain and discomfort. Patients' prognosis was excellent, with a 97.1% 5-year DSS rate and a 95.7% 5-year OS rate in SEER cohort. These data basically consisted of the information reported in the former literature [11, 17]. In comparison to tumors located in other sites, patients in the HOP group were younger than those in the OOP group. Tumor location determined the surgical approach and also influenced the number of resected lymph nodes. Compared with the OOP group, HOP patients had longer operation time and hospital stay. What is more, patients with tumors which developed in the head of the pancreas had a worse prognosis according to our survival analysis. To our knowledge, this is the first study to identify the clinicopathological characteristics related to pancreatic head SPNs.

In public mind, SPN is a kind of cystic, solid, and localized tumor with an excellent prognosis [2]. The treatment of SPN is complete resection no matter with or without metastasis. Local resection or enucleation is employed for small tumors with complete capsule; pancreatic head tumor is treated with pancreatoduodenectomy (Whipple procedure); distal pancreatectomy is performed for pancreatic body and/or tail tumors [18]. For SPN patients in different situations, in addition to these location-related basic rules, there are no clear rules and differences for surgical methods and follow-up examinations. However, there are still some SPNs which may behave aggressively and lead to poor ending. Yin et al. reported that large tumor size, pancreatic tail localization, and disrupted capsule were viewed as the malignant SPN phenotype [19]. Mao considered that SPNs in older and male patients were more aggressive [20]. In our research, tumor size, gender, pancreatic tail location, and age have no significant correlation to the patients' prognosis. Pancreatic head location seems more worthy of our attention. Goh et al. reported that age, sex, tumor size, elevated CEA, and elevated CA-199 could not predict the malignancy of SPN before surgery, and our results were largely similar to the conclusion of Goh's data [21]. Researchers supposed that pancreatic head SPNs could lead to the apparent clinical symptoms which increased the chances of early detection and thus avoid further tumor progression [19]. However, in our study, there was no significant difference in the clinical symptoms between different tumor sites (*p* = 0.979) and the prognosis was worse in pancreatic head SPN patients which did not support the abovementioned theory. We infer poor prognosis of SPNs in the pancreatic head may be due to the following reasons.

From the anatomical standpoint, the head of the pancreas is rich in blood supply and surrounded by many lymph nodes, which facilitates the early metastasis of SPN. Pancreatic head and descendant duodenum are closely linked, which provides the direction for the invasion of SPN. From the perspective of embryonic development, pancreatic head originates from the ventral pancreas, and the other parts originate from the dorsal pancreas. It can also partly explain the differences in the clinicopathological features and outcomes of SPNs at different tumor locations. In terms of surgical treatment, pancreatic head SPNs are primarily treated by pancreatoduodenectomy. Due to the difficulty of its operation procedures, the probability of intraoperative tumor implantation and postoperative complications would be increased, and the prognosis of patients will be influenced accordingly.

For the above reason, the treatment and follow-up of pancreatic head SPNs should be more cautious. Complete surgical resection, with negative surgical margins, needs to be applied in these patients. Appropriate lymphadenectomy is necessary to prevent the nodal metastases, and the follow-up period of these patients should be extended appropriately. The limitations to this study include the small number of cases and the retrospective nature. Long-term follow-up is necessary because of the indolent character of SPN. The patients are predominantly yellow and white, and extensive multicenter studies with more races are needed to identify the risk factors associated with the SPN patients' outcome. What is more, due to the reason of data collection, the roles of pathology results, radiotherapy, and chemotherapy have not been identified in our study. More prognostic factors such as tumor calcification, solid component, capsule integrity, and IHC markers need to be explored in further research.

## 5. Conclusion

SPNs are rare tumors with good prognosis. Tumor sites are associated with the prognosis of SPN patients. Patients with pancreatic head SPNs have worse outcome and accompany distinct clinicopathological features.

## Figures and Tables

**Table 1:** Clinicopathological features of the SPN patients in our study.

Characteristics	Our cohort	Characteristics	SEER cohort
	Numbers (percentage)		Numbers (percentage)
Age	29 (16–45)	Age	33 (22–45)
Sex		Sex	
Male	12 (21.8)	Male	23 (16.3)
Female	43 (78.2)	Female	118 (83.7)
Location		Location	
Head	24 (42.9)	Head	35 (24.8)
Body	9 (16.1)	Body	19 (13.5)
Tail	11 (19.6)	Tail	69 (48.9)
Others	11 (19.6)	Others	18 (12.8)
Size	5.0 (4.0–7.0)	Size	5.4 (3.5–9.5)
Operation category		Operation category	
1	3	1	5 (3.5)
2	33	2	81 (57.4)
3	18	3	22 (15.6)
4	1	4	29 (20.6)
Symptom		Race	
Pain	22 (40)	White	101 (71.6)
Asymptomatic	26 (47.3)	Yellow	16 (11.3)
Mass	5 (9.1)	Black	20 (14.2)
Others	2 (3.6)	Lymph nodes examined	
Complication arise	31 (57.4)	≤5	65 (47.8)
Operation time	330 (270–435)	>5	71 (52.2)
Postoperative diet days	6 (3.5–9)		
Discharge days	12 (10.5–19)		
Hospital stay, days	17 (13.5–31)		

Values are expressed as number (percentage) or median (Q1–Q3). Operation category: 1: local excision of tumor; 2: partial pancreatectomy and partial or local pancreatectomy and duodenectomy without distal/partial gastrectomy; 3: Whipple's procedure and extended pancreatoduodenectomy; and 4: other operation methods.

**Table 2:** Survival data of 188 SPN cases in our study.

Our cohort		SEER cohort	
Survival characteristics	Parameter	Survival characteristics	Parameter
Follow-up time		Follow-up time	
Mean (m ± SD)	33.7 ± 18.7	Mean (m ± SD)	58.3 ± 51.3
Median (m, range)	28 (9–65)	Median (m, range)	50 (2–152)
Survival data		Survival data	
All-cause deaths	5	SPN-related deaths	5
Survival rates (%)		All-cause deaths	9
1/3 OS	95.7/89.4	Survival rates (%)	
1/3 HOP-OS	92.3/88.5	1/3/5 DSS	98.5/97.8/97.1
1/3 OOP-OS	100/90.5	1/3/5 OS	97.8/97.2/95.7

**Table 3:** Prognostic factors for DSS and OS in SEER cohort according to univariate and multivariate analysis.

Survival	Univariate analysis				Multivariate analysis			
DSS	HR	Down	Up	*p* value	HR	Down	Up	*p* value
Race	0.54	0.10	2.89	0.47				
Age	1.02	0.96	1.08	0.62				
Gender	1.22	0.14	11.04	0.86				
Location	13.99	1.56	125.33	0.02	12.75	1.31	124.21	0.028
Size	1.009	0.959	1.063	0.719				
Operation methods	1.49	0.58	3.78	0.41				
Lymph nodes examined	1.04	0.98	1.11	0.23				

OS	HR	Down	Up	*p* value	HR	Down	Up	*p* value
Race	0.78	0.29	0.78	0.63				
Age	1.03	0.99	1.08	0.17				
Gender	1.35	0.28	6.55	0.71				
Location	4.04	1.08	15.07	0.038	6.08	1.29	28.66	0.022
Size	1.01	0.94	1.08	0.89				
Operation methods	1.42	0.70	2.87	0.33				
Lymph nodes examined	1.03	0.97	1.09	0.31				

DSS: disease-specific survival; OS: overall survival; HR: hazard ratio.

**Table 4:** Comparison of selected clinicopathological parameters between the HOP group and the OOP group.

Our cohort				SEER cohort			
Character	HOP	OOP	*p* value	Character	HOP	OOP	*p* value
*Gender*			0.069	Gender			0.496
Male	15	20		Male	7	16	
Female	44	117		Female	28	90	
*Operation*			<0.001	Operation			<0.001
1	0	3		1	3	2	
2	5	28		2	11	70	
3	18	0		3	15	7	
4	1	0		4	5	24	
*Age*			0.007	Age			0.647
Juvenile	11	4		Juvenile	3	12	
Adult	13	27		Adult	32	94	
*Symptom*			0.979	Race			0.152
Pain	9	13		White	25	76	
By accident	12	14		Yellow	6	10	
Mass	2	3		Black	2	18	
Others	1	1					
*Complication*			0.667				
No	11	12					
Yes	13	18					
*Age* (year)	26.21 (18.08)	36.61 (16.89)	0.03	Age (year)	30.57 (12.94)	37.01 (15.77)	0.03
*Size* (cm)	5.50 (3.05)	6.50 (3.72)	0.29	Size (cm)	6.54 (3.29)	7.64 (10.09)	0.55
*Operation time* (minute)	371.74 (121.61)	228.87 (71.62)	<0.001	Examined lymph nodes	11.74 (9.53)	7.10 (8.17)	0.01
*Examined lymph nodes*	8.20 (5.02)	6.27 (5.53)	0.36				
*Restoration oral feeding* (day)	7.93 (6.40)	4.82 (2.70)	0.10				
*Postoperative discharge* (day)	18.96 (16.98)	13.52 (5.70)	0.10				
*Hospital stay* (day)	27.46 (18.58)	19.03 (7.89)	0.05				

## Data Availability

All data used to support the findings of this study are available from the corresponding author upon request.
